# Effect of Endovenous Laser Ablation Along With Compression Therapy on Chronic Venous Ulcer Healing

**DOI:** 10.7759/cureus.33406

**Published:** 2023-01-05

**Authors:** Sheetal Uttaray, Venkata Vineeth Vaddavalli, Ajay Savlania, Arunanshu Behera, Lileswar Kaman, Ujjwal Gorsi

**Affiliations:** 1 Department of General Surgery, Postgraduate Institute of Medical Education and Research, Chandigarh, IND; 2 Department of Radiology, Postgraduate Institute of Medical Education and Research, Chandigarh, IND

**Keywords:** healing, compression therapy, endovenous ablation, venous ulcer, chronic venous disease

## Abstract

Introduction

Chronic venous insufficiency is a major cause of morbidity, and there is a paucity of data regarding its epidemiology due to the lack of a central wound registry. In this study, we aimed to study the time for healing of the ulcer and compliance with compression therapy (CT) following endovenous laser ablation (EVLA) ± ultrasound-guided foam sclerotherapy (UGFS) along with CT in patients with chronic venous ulcers.

Methods

This prospective observational study was conducted from January 2020 to June 2021 after obtaining institutional ethical committee clearance. Patients with chronic venous ulcers (>six weeks to <six months duration) were included in this study. Demographic details, venous duplex findings, and duration of ulceration were noted. All patients underwent EVLA (Biolitec^®^ 1470 nm) ± UGFS and CT. The patients were followed up weekly till the healing of the ulcer, followed up at one month, three months, and then at six monthly intervals to look for recurrence. Venous clinical severity score (VCSS) at presentation and follow-up, compliance with CT, rate of ulcer healing at six months, and recurrence rates at two years of follow-up were observed prospectively.

Results

The mean age of the study participants was 45.7±14.2 years, of which 42 (84%) were males. Ulcer size of <2 cm was present in 38%, 2-4 cm in 52%, and >4 cm in 10% of patients. A total of 38% of patients underwent only EVLA, and EVLA+UGFS were done in 62% of participants. The healing rate at six months follow-up was 92%, with the average time taken being 2.55±1.38 months. Those who remained with an unhealed ulcer at six months follow-up had an ulcer size of >5 cm and an age of >50 years. Ninety-six percent of the patients were compliant with CT after an endovenous intervention. The recurrence rate at two years post-ablation was 6%. VCSS was 19.66±3.23 at presentation and 5.5±2.82 at six months of follow-up.

Conclusion

Endovenous ablation of superficial venous reflux along with CT is associated with a shorter healing time of venous ulcerations and reduced chances of recurrence. There is an improvement in VCSS score over the period of six months follow-up.

## Introduction

Chronic venous leg ulceration is the most severe manifestation of chronic venous insufficiency (CVI), accounting for most cases of lower limb ulcerations. The American Venous Forum (AVF) defined venous ulcerations as “a full-thickness defect of the skin, most frequently in the ankle region, that fails to heal spontaneously and is sustained by chronic venous disease, which is based on duplex ultrasound testing” [1]. The prevalence of venous ulcerations among adults aged between 18 and 64 years was around 1%, with prevalence increasing with age [2]. Venous ulcerations show difficulty in healing and are a leading cause of morbidity, leading to lost productivity at a young age and increased impairment in the elderly. Its treatment is expensive, leading to a substantial economic burden on the healthcare system [3]. It was shown that the key determinants in the healing of venous ulcers are location, duration, and the area around the ulcer [4].

Minimally invasive procedures like endovenous laser ablation (EVLA), radiofrequency ablation (RFA), and ultrasound-guided foam sclerotherapy (UGFS) have largely replaced traditional surgeries in treating CVI. The AVF and the Society for Vascular Surgeons (SVS) have recommended that EVLA and RFA be equally safe and effective [5]. Patient education and compression therapy play a vital role in preventing recurrence [6]. Various studies have shown early intervention, along with compression therapy, showed higher healing rates and decreased recurrence compared to compression therapy alone [7,8]. The data regarding the healing patterns of chronic venous ulcers in the Indian population are limited. Hence, we conducted this study to assess the time for healing of the ulcer and compliance with compression therapy following EVLA±UGFS along with compression therapy in patients with chronic venous ulcers in the Indian subcontinent.

## Materials and methods

This prospective observational study was conducted from January 2020 to June 2021 at a tertiary care center in Northern India after obtaining institutional ethical committee clearance (#NK/5907/MS/040). Written informed consent is obtained from all the study participants. Patients with chronic venous ulcers (>six weeks to <six months duration) were included in this study. Patients with a presence or history of deep vein thrombosis (DVT), ankle-brachial index (ABI) less than 0.8, and age less than 18 years were excluded. The demographic profiles and the comorbidities of the study participants were noted during their initial visit to the clinic. All underwent clinical examination to look for the characteristics of the ulcer and the area surrounding it. The venous duplex examination was done to look for reflux in the superficial venous system, deep venous system, or the perforators and to rule out DVT. All the patients underwent EVLA (Biolitec® 1470 nm) ± UGFS and compression therapy. For the saphenofemoral junction incompetence, ablation of the great saphenous vein was done, and for saphenopopliteal junction incompetence, the short saphenous vein was ablated. A 0.5% sodium tetradecyl sulfate was used as a sclerosant, and foam was created using air and sclerosant in a 1:4 ratio by the Tessari method. The time from the onset of the ulcer to the intervention was noted. The patients were followed up weekly till the healing of the ulcer, followed up at one month, three months, and then at six monthly intervals to look for recurrence. The mean duration for the healing of the ulcer and the recurrence rates at one year and two years of follow-up were assessed. Venous clinical severity score (VCSS) was used to determine the response to the treatment (see Appendix). It was calculated at the time of presentation and one month, three months, and six months after the intervention. The compression therapy was continued after healing in all the patients, and compliance with the compression therapy was noted. The data regarding the study participants was maintained in an electronic case record form.

Statistical analysis was done using Microsoft Excel and Statistical Package for the Social Sciences (SPSS) software version 23 (Chicago, IL: IBM Corp.). All quantitative variables were described with measures of central tendency (mean and median) and measures of dispersion (standard deviation and standard error). Categorical variables were expressed as frequencies and proportions.

## Results

**Figure 1:**
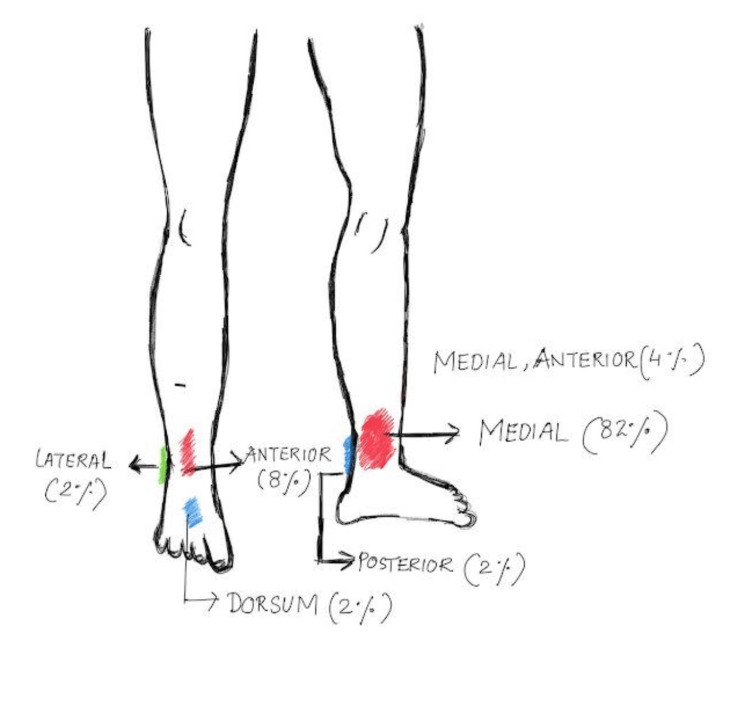
Distribution of ulcers at various sites. The image is created by the authors of this study.

A total of 50 patients were included in this study. The median age was 44 years. Demographic profile, comorbidities, and clinical presentation are shown in Table 1. A total of 84% were males, and 30% were smokers. The ulcers were present more on the left lower limb than on the right (54% vs. 46%). The majority of the ulcers were presented in the gaiter’s area (in the anterior part of the leg between the knee and malleoli), and the frequency of distribution of ulcers at various sites is shown in Figure 1. The mean duration of the ulcer till intervention was 3.9±2.3 months, and a majority of the ulcers had a size between 2 and 4 cm (52%).

**Table 1:** Demographic profile of the study cohort.

Parameter		N (%)
Sex	Male	42 (84%)
	Female	8 (16%)
Comorbidities	Smoking	15 (30%)
	Hypertension	13 (26%)
	Diabetes mellitus	10 (20%)
	Coronary artery disease	4 (8%)
Clinical presentation	History of prolonged standing	41 (82%)
	Pain in the affected limb	33 (66%)
	Edema	37 (74%0
	Lipodermatosclerosis	32 (64%)
Ulcer size	<2 cm	19 (38%)
	2-4 cm	26 (52%)
	>4 cm	5 (10%)

**Figure 2:**
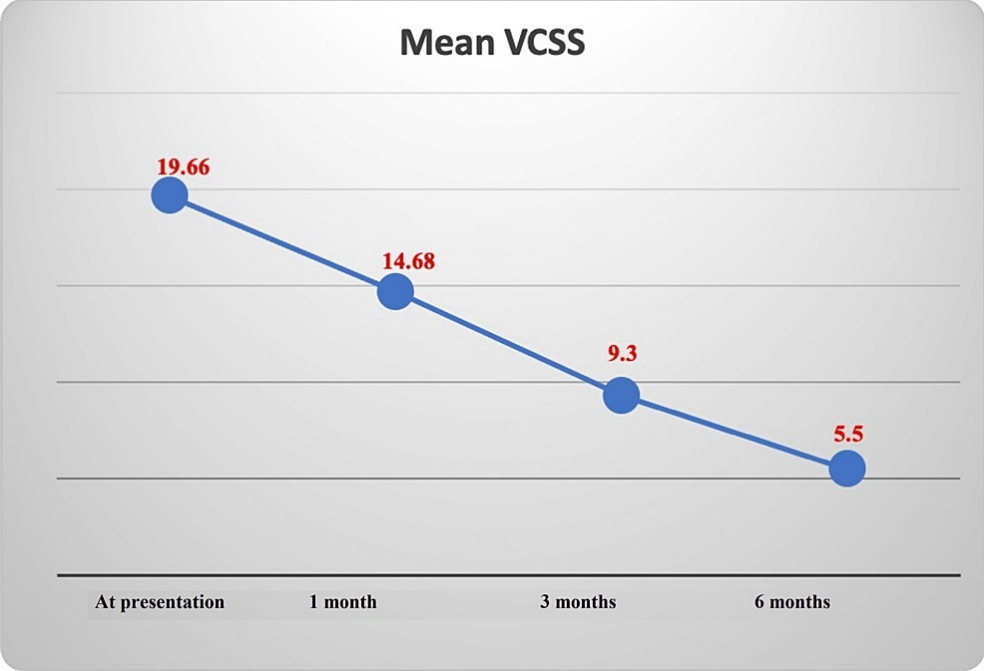
Venous clinical severity score in the follow-up period. VCSS: venous clinical severity score

Venous duplex findings are shown in Table 2. Most patients had both saphenofemoral junction and perforator incompetence (42%). Only EVLA was performed in 19 patients (38%), and EVLA with UGFS was done in 31 patients (62%). At six months of follow-up, 46 (92%) patients achieved complete healing of the ulcer. Cumulative healing rates at one month, three months, and six months of follow-up were 26%, 80%, and 92%, respectively. The average time taken for healing of the ulcer was 2.55±1.38 months. The recurrence rate of the ulcer at two years of follow-up was 6%. The characteristics of patients with and without recurrence are shown in Table 3. There was a decline in the VCSS over the duration of follow-up, implying the improvement of the symptoms in the affected limbs. The progression of the VCSS over six months is shown in Figure 2. Forty-eight out of 50 patients showed moderate compliance with compression therapy.

**Table 2:** Venous duplex findings at presentation in the study cohort. SFJ: saphenofemoral junction; SPJ: saphenopopliteal junction

Finding	N (%)
SFJ incompetence only	5 (10%)
Perforator incompetence only	8 (16%)
SFJ+perforator incompetence	21 (42%)
SPJ+perforator incompetence	2 (4%)
SFJ+SPJ+perforator incompetence	12 (24%)
SFJ+SPJ incompetence	2 (4%)
Deep vein incompetence	2 (4%)

**Table 3:** Comparison between recurrent and non-recurrent ulcers. VCSS: venous clinical severity score

Variable	Recurrence	No recurrence
Number of patients	3	46
Age (in years ± SD)	59.6±10.7	45±14.1
Size of ulcer (cm)	4±1	2.2±1.65
Duration of ulcer (months)	6	3.8±2.3
VCSS at presentation	18.6±3.05	14.2±3.9

## Discussion

Chronic venous insufficiency with ulcerations over lower limbs is commonly seen in our day-to-day practice. They are a common cause of pain and impaired quality of life. In western countries, the prevalence of CVI ranges from 3% to 7% in females and 2% to 7% in males [9]. However, there is a lack of data regarding the epidemiology of CVI and venous ulcers due to the lack of a central wound registry and quality research in this field. Several studies have shown compressive therapy can achieve successful healing of ulcerations in 70% or more patients in six months [7,10]. However, the recurrence rate is high, up to 25% after one year [11]. Bonn Vein Study showed that 68% of patients were non-compliant with the therapy due to tight wrapping [12]. Also, many patients tend to discontinue compression therapy once the ulcer heals, which can lead to recurrence. The application of compression bandaging is challenging, especially for the elderly with limited dexterity [13]. In our study, 96% of the patients were compliant with compression therapy.

It is suggested that surgical correction of superficial incompetence can have clinical benefits in healing ulcers. The “effect of surgery and compression on healing and recurrence” (ESCHAR) trial provided convincing evidence that surgical correction of superficial venous incompetence reduced ulcer recurrence and improved ulcer-free time compared to compression therapy [7]. The surgical treatment of superficial venous reflux disease of lower limbs included high ligation, stripping the vein to the knee level, and phlebectomy. However, surgery is an invasive procedure associated with morbidities, including wound infection, saphenous nerve injury, deep vein thrombosis, and recurrence. Recently, minimally invasive techniques such as EVLA, RFA, and UGFS have replaced the surgical stripping of saphenous veins as the primary treatment of superficial reflux disease. Endovenous thermal ablation has been shown to have high rates of saphenous venous closure with low recanalization of the treated vein in long-term follow-ups [14].

The median age of the patients in our study is 44 years. Compared to the epidemiological study by Baker et al., in which the prevalence of ulcers increased with age and maximum in persons with age >70 years, patients in our study belonged to age groups 30-45 years [15]. In most studies, chronic venous ulcers were reported to be more common in women, but in our study, 42 (84%) were males [16]. This may be due to the occupational and socioeconomic differences of the patients attending our hospital. According to a study by Gourgou, smoking is significantly associated with chronic venous insufficiencies [17]. A total of 30% of the patients in our study were smokers.

The majority of the patients in our study had an ulcer size of 2-4 cm (52%), and 10% had a size of more than 4 cm. The mean duration of the ulcer was 3.9 months. Seventy percent had superficial and perforator reflux, 14% had only superficial reflux, 12% had only perforator reflux, and 4% had all superficial, deep, and perforator reflux disease, according to a venous duplex study. In 1991, Hanrahan et al. studied the distribution of valvular incompetence in patients with stasis venous ulcerations. They found multisystem incompetence in 66.3% (superficial+perforator 19%, superficial+deep 11.6%, perforator+deep 4.2%, superficial+deep+perforator 31.6%), isolated superficial 16.8%, isolated perforator 8.4%, isolated deep vein 8.4%, and no incompetence in 6.3% [18].

Two main objectives in treating chronic venous ulcerations are healing of the lesion and prevention of recurrence. When treatment is inappropriate, 30% of healed ulcers recur in the first year and 78% after two years [19]. In our study, 38% of patients underwent only EVLA, while 62% underwent EVLA along with USG-guided foam sclerotherapy. The mean ulcer healing time was 2.55±1.38 months. The cumulative healing rate at one, three, and six months were 26%, 80%, and 92%, respectively. The recurrence of ulceration was seen in one patient (2%) after one year and three patients (6%) after two years of surgery. In a study by Marston et al., ulcer healing occurred in 57% patients at three months, 74% patients at six months, and 79% patients at 12 months after endovenous ablation of limbs with chronic ulcers [14]. In the early venous reflux ablation (EVRA) trial, the time for ulcer healing for patients who underwent early endovenous ablation along with compression therapy was 56 days (95% CI 49-66 days), and for patients who received compression-only with deferred endovenous intervention until six months if the ulcer remained unhealed was 82 days (95% CI 69-92). The recurrence rate during one year follow-up period was 11.4% in the early intervention group and 16.5% in the deferred intervention group [8]. In a study by Alden et al., ulcerations treated with compression therapy were compared with ulcerations treated with both compression and minimally invasive techniques (intervention group). Ulcers in the intervention group healed faster than in the compression group (7.9 weeks vs. 22 weeks, p<0.001). The ulcer recurrence rate was significantly higher in the compression group compared to the intervention group (48.9% vs. 22.9%, p=0.004) [20]. Our findings are similar to the above studies and prove that correction of underlying pathology, i.e., local venous hypertension, by minimally invasive techniques like endovenous ablation and UGFS, allows faster healing of ulcers and prevents recurrence.

The VCSS was developed to objectively evaluate the response of patients with CVI to the treatment as it can identify subtle changes in the subject over time. Meissner et al. validated this scoring system and showed low interobserver variation, with a reliability coefficient of 0.6 [21]. In our study, the mean score on venous clinical severity score at presentation was 19.66±3.23, which showed gradual improvement in the subsequent follow-up period with the score of 14.68±4.08, 9.3±4.04, 5.5±2.8 at one, three, and six months post-endovenous ablation, respectively. These findings are similar to the results of the EVRA trial, in which the mean scores on the VCSS assessment tool at randomization were 15.8±3.3 in the early intervention group and 15.7±3.1 in the deferred intervention group. At six weeks, it was 10.5±4.7 in the early intervention group and 12.6±4.4 in the deferred intervention [8]. This shows that EVLA, along with compression therapy, leads to symptomatic improvement in patients with venous ulcers.

## Conclusions

Endovenous ablation of superficial venous reflux and compression therapy are associated with a shorter healing time of venous ulcerations and reduced chances of recurrence. As endovenous intervention can be done in a one-time procedure, its success is less dependent on patients’ compliance than it would be with compression therapy. A reasonable rate of venous ulcer healing can be achieved with EVLA and compression therapy, even in an Indian scenario where high temperature compared to Western Countries is a deterrent factor for patients to comply optimally with compression therapy.

## Appendices

Venous clinical severity score used in the study

**Table 4:** Venous clinical severity score used in the study. Absence of venous disease is defined by a score of ≤3 and a score ≥8 defines severe disease.

Attribute	Absent (0)	Mild (1)	Moderate (2)	Severe (3)
Pain	None	Occasional	Daily not limiting daily activities	Daily interfering with daily activities
Varicose veins (>4 mm)	None	Few, scattered	Confined to calf or thigh	Involves calf and thigh
Venous edema	None	Limited to foot and ankle	Extends above the ankle but below knee	Extends to knee and above
Skin pigmentation	None	Limited to perimalleolar area	Diffuse over lower third of calf	Wider distribution above lower third with recent pigmentation
Inflammation	None	Limited to perimalleolar area	Diffuse over lower third of calf	Severe cellulitis or significant venous eczema
Induration	None	Limited to perimalleolar area	Diffuse over lower third of calf	Entire lower third of leg or more
Number of active ulcers	0	1	2	>2
Longest active ulcer duration	N/A	<3 months	>3 months but <1 year	Not healed for 1 year
Largest active ulcer size (cm)	N/A	Diameter <2 cm	Diameter 2-6 cm	Diameter >6 cm
Ulcer duration	None	<3 months	3-12 months	>1 year
Compression stocking	None	Intermittent use	Most days	Full compliance

## References

[1] O'Donnell TF Jr, Passman MA, Marston WA (2014). Management of venous leg ulcers: clinical practice guidelines of the Society for Vascular Surgery® and the American Venous Forum. J Vasc Surg.

[2] Ruckley CV, Evans CJ, Allan PL, Lee AJ, Fowkes FG (2002). Chronic venous insufficiency: clinical and duplex correlations. The Edinburgh Vein Study of venous disorders in the general population. J Vasc Surg.

[3] Lal BK (2015). Venous ulcers of the lower extremity: definition, epidemiology, and economic and social burdens. Semin Vasc Surg.

[4] Marston WA, Ennis WJ, Lantis JC 2nd (2017). Baseline factors affecting closure of venous leg ulcers. J Vasc Surg Venous Lymphat Disord.

[5] Dietzek AM (2007). Endovenous radiofrequency ablation for the treatment of varicose veins. Vascular.

[6] Motykie GD, Caprini JA, Arcelus JI, Reyna JJ, Overom E, Mokhtee D (1999). Evaluation of therapeutic compression stockings in the treatment of chronic venous insufficiency. Dermatol Surg.

[7] Gohel MS, Barwell JR, Taylor M (2007). Long term results of compression therapy alone versus compression plus surgery in chronic venous ulceration (ESCHAR): randomised controlled trial. BMJ.

[8] Gohel MS, Heatley F, Liu X (2018). A randomized trial of early endovenous ablation in venous ulceration. N Engl J Med.

[9] Callam MJ (1994). Epidemiology of varicose veins. Br J Surg.

[10] Barwell JR, Davies CE, Deacon J (2004). Comparison of surgery and compression with compression alone in chronic venous ulceration (ESCHAR study): randomised controlled trial. Lancet.

[11] Nicolaides A, Kakkos S, Baekgaard N (2018). Management of chronic venous disorders of the lower limbs. Guidelines according to scientific evidence. Part I. Int Angiol.

[12] Maurins U, Hoffmann BH, Lösch C, Jöckel KH, Rabe E, Pannier F (2008). Distribution and prevalence of reflux in the superficial and deep venous system in the general population--results from the Bonn Vein Study, Germany. J Vasc Surg.

[13] Sharif MA, Lau LL, Lee B, Hannon RJ, Soong CV (2007). Role of endovenous laser treatment in the management of chronic venous insufficiency. Ann Vasc Surg.

[14] Marston WA, Crowner J, Kouri A, Kalbaugh CA (2017). Incidence of venous leg ulcer healing and recurrence after treatment with endovenous laser ablation. J Vasc Surg Venous Lymphat Disord.

[15] Baker SR, Stacey MC, Jopp-McKay AG, Hoskin SE, Thompson PJ (1991). Epidemiology of chronic venous ulcers. Br J Surg.

[16] Beaglehole R (1986). Epidemiology of varicose veins. World J Surg.

[17] Gourgou S, Dedieu F, Sancho-Garnier H (2002). Lower limb venous insufficiency and tobacco smoking: a case-control study. Am J Epidemiol.

[18] Hanrahan LM, Araki CT, Rodriguez AA, Kechejian GJ, LaMorte WW, Menzoian JO (1991). Distribution of valvular incompetence in patients with venous stasis ulceration. J Vasc Surg.

[19] Mayer W, Jochmann W, Partsch H (1994). Varicose ulcer: healing in conservative therapy. A prospective study. [Article in German]. Wien Med Wochenschr.

[20] Alden PB, Lips EM, Zimmerman KP (2013). Chronic venous ulcer: minimally invasive treatment of superficial axial and perforator vein reflux speeds healing and reduces recurrence. Ann Vasc Surg.

[21] Meissner MH, Natiello C, Nicholls SC (2002). Performance characteristics of the venous clinical severity score. J Vasc Surg.

